# Leiomyoma in Vulva: A Diagnostic Dilemma

**DOI:** 10.1155/2014/386432

**Published:** 2014-02-10

**Authors:** Deeksha Pandey, Jyothi Shetty, Aashish Saxena, P. S. Srilatha

**Affiliations:** ^1^Department of OBGYN, KMC, Manipal University (MU), Manipal 576104, India; ^2^KMC, MU, Manipal 576104, India; ^3^Department of Pathology, MMMC, MU, Manipal 576104, India

## Abstract

With the help of this case we summarize some crucial features to be picked up from history and examination before labeling a case as Bartholin's abscess or cyst. A 20-year old unmarried girl, deaf and mute since birth, was initially diagnosed to have Bartholin's abscess. On careful reexamination after inflammation subsided, a decision of excision of this tumor was taken. Intraoperatively it was found to be well encapsulated. Histopathology ascertained the diagnosis of vulval leiomyoma.

## 1. Introduction

Unilateral inflamed vulval swelling in a woman of reproductive age is commonly Bartholin's abscess. We report here an interesting case of vulval leiomyoma which was initially misdiagnosed as Bartholin's abscess. We summarize some crucial features to be picked up from history and examination before labeling a case as Bartholin's abscess or cyst.

## 2. Case Report

**Figure 1:**
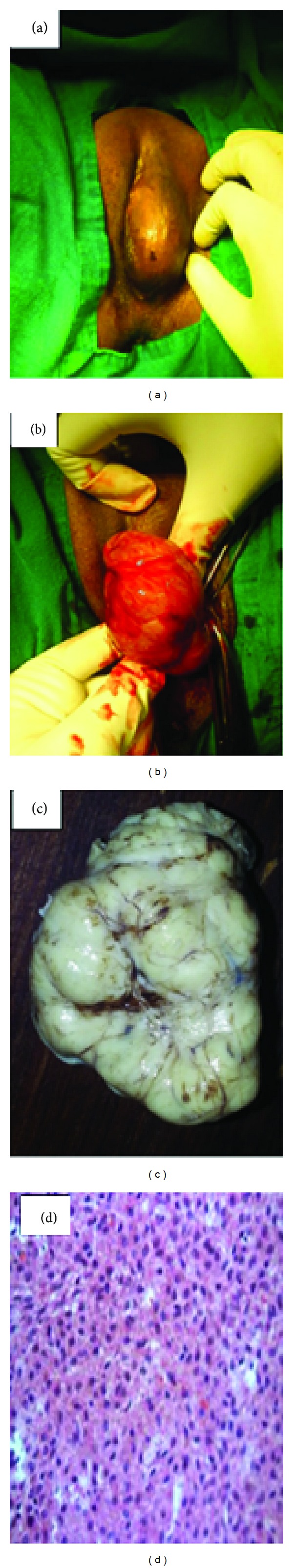
(a) Vulval swelling after the inflammation subsided. (b) Intraoperative: enucleation. (c) Gross specimen consisted of single, nodular, grey-white tissue mass weighing 56 gm; measures 6 × 4 × 3 cm. (d) Microscopy showing a benign tumor composed of sheets and fascicles of oval to spindle shaped cells with abundant dense cytoplasm, microcystic areas, areas of hyalinization, and focal lymphocytic infiltrate.

A 20-year old unmarried girl, deaf and mute since birth, presented with swelling in vulva for 6 months. The swelling which was slowly increasing in size had become extremely painful for the last one week. There was no history of fever and vaginal discharge. On examination her general condition was fair and vital signs were stable. She was afebrile and there was no inguinal lymphadenopathy. Local examination revealed a labial swelling on the left side with signs of inflammation. Diagnosis of Bartholin's abscess was made and she was started on parenteral antibiotics. Within 24 hours her pain decreased and inflammation subsided. As the girl and her parents insisted that she was sexually naïve, she was reexamined. On reexamination 5 × 5 cm swelling on the left labia majora was noted. There were no signs of inflammation now. The swelling felt firm in consistency (not cystic). Labia minora was not everted and hymen was intact (Figure 1(a)). Keeping all these facts in mind a decision of surgical excision of the swelling was taken. Under spinal anesthesia a 3 cm incision was made at the mucocutaneous junction and a firm encapsulated mass was enucleated after dissection along its capsular plane (Figure 1(b)). Base was obliterated with interrupted sutures and overlying skin incision was closed.

On histopathological examination grossly the specimen consisted of single, nodular, grey-white tissue mass weighing 56 g that measured 6 × 4 × 3 cm (Figure 1(c)). On cut section there were grey-white areas, focal cystic areas, and focal myxoid areas, with specks of hemorrhage. Microscopy revealed benign tumor composed of sheets and fascicles of oval to spindle shaped cells with abundant dense cytoplasm, microcystic areas, areas of hyalinization, and focal lymphocytic infiltrate (Figure 1(d)). Thus final diagnosis of epitheliod leiomyoma was ascertained.

## 3. Discussion 

**Figure 2:**
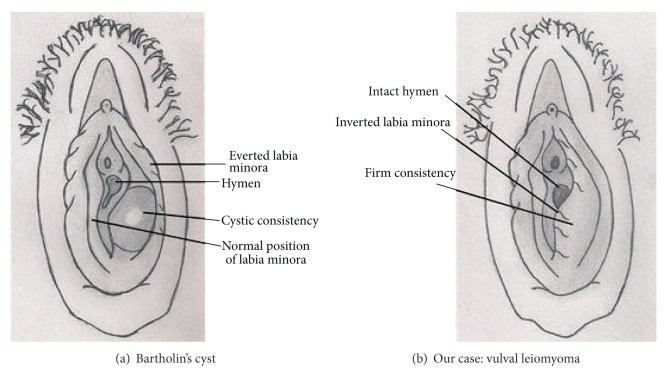
Crucial features to look for in a case of vulval swelling.

This case emphasizes the importance of detailed history and meticulous examination. Bartholin's cyst or abscess is commonly seen in sexually active women. Any case diagnosed initially as Bartholin' abscess needs to be reexamined once the inflammation subsides. Inverted labia minora, firm consistency, and intact hymen point towards reconsidering the diagnosis (Figure 2). Smooth muscle tumors though rare but do occur in the vulva. Transperineal ultrasonography is of help in establishing the diagnosis. Surgical excision is the treatment.

Leiomyoma or fibroid though very commonly seen in the uterus is a rare entity in vulva, ovaries, urethra, and urinary bladder. It is interesting to note that it may arise in nearly any anatomic site [1].

Recently Youssef et al. reported a case of 39-year-old, P2L2 lady who had 2 to 3 cm solid mass on her external genitalia for 4 years enlarged to 15 cm in 6 months. This lesion was excised. On histopathological examination it was diagnosed as leiomyoma of vulva [2]. Another case was reported in a 56-year-old, postmenopausal woman, where the initial suspicion was of Bartholin's gland carcinoma [3]. On initial presentation most of the vulval leiomyoma are usually misdiagnosed as Bartholin's cyst or abscess [2–4].

Nielsen et al. studied 25 cases of leiomyoma of vulva. Most patients in their series presented with a painless mass, other symptoms being pain, itching, and erythema. Most common preoperative diagnosis was Bartholin's gland cyst. There were 20 leiomyoma (4 atypical) and 5 leiomyosarcoma. On followup 4 leiomyosarcomas recurred and one of these women died. Only 1 patient with leiomyoma had recurrence after 10 years. According to them major diagnostic problem with smooth-muscle tumors of the vulva is the distinction between benign and malignant forms [4]. In 1965 an interesting case of leiomyoma of vulva and coexisting leiomyoma of esophagus was reported in a mother and her daughter from Helsingborg, Sweden [5]. Later similar syndrome was reported by others too [6–9]. Very recently Tian et al. reported a case of 64-year-old female (phenotypically) with androgen insensitivity syndrome having bilateral vulval leiomyoma [10].

Transperineal ultrasonography is of help in establishing the diagnosis of vulval leiomyoma while MRI is of help in differentiating benign and malignant forms in doubtful cases. A characteristic finding of low signal intensity mimicking that of smooth muscle on T2-weighted images is the key to diagnosis. The MR signal in the tumors is isointense to that in muscle on T1-weighted images, and the tumors enhance homogeneously after the administration of contrast material [1].

Surgical excision is the treatment of choice in all smooth muscle tumors of vulva. Histopathological confirmation of its benign or malignant nature is mandatory. This differentiation is a great challenge to the pathologist. Long term followup of all cases is advisable.

## 4. Conclusion

Extrauterine leiomyomas are rare, and they present a greater diagnostic challenge. Unusual sites of origin include the vulva, ovaries, urinary bladder, and urethra. Other rare locations are sinonasal cavities, orbits, kidneys, and skin. Differential diagnosis includes Bartholin's cysts, fibromas, lymphangiomas, soft-tissue sarcomas, and neurogenic tumors. Ultrasonography/MRI might be of help in establishing the diagnosis. Labial leiomyomas are treated with conservative surgery. After the surgery long-term follow up is advised.
